# Extra-large pore JZO zeolites with tunable Si/Al ratios as efficient catalysts for degradable plastic monomer production

**DOI:** 10.1039/d6ra01852a

**Published:** 2026-06-01

**Authors:** Shuman Gao, Xinxin Wang, Shaolei Gao, Mohammad Fahda, Dongyue Wang, Haijun Yu, Zijian You, Xiuning Liu, Liang Qi, Feng Shao, Peng Lu, Valentin Valtchev

**Affiliations:** a Key Laboratory of Marine Chemistry Theory and Technology of Ministry of Education, College of Chemistry and Chemical Engineering, Ocean University of China Qingdao 266100 China feng.shao@88.com; b The ZeoMat Group, Key Laboratory of Photoelectric Conversion and Utilization of Solar Energy, Qingdao New Energy Shandong Laboratory, Qingdao Institute of Bioenergy and Bioprocess Technology, Chinese Academy of Sciences 266101 Qingdao China lupeng@qibebt.ac.cn; c National Engineering Research Center of Lower-Carbon Catalysis Technology, Dalian National Laboratory for Clean Energy, Dalian Institute of Chemical Physics, Chinese Academy of Sciences Dalian 116023 Liaoning China; d Normandie University, ENSICAEN, UNICAEN, CNRS, Laboratoire Catalyse et Spectrochimie F-14000 Caen France valentin.valtchev@ensicaen.fr

## Abstract

JZO zeolite, the first stable aluminosilicate extra-large-pore zeolite, bridges the long-standing gap between microporous and mesoporous zeolites, offering highly accessible pore systems that are beneficial for molecular diffusion, active-site accessibility, and the processing of bulky molecules. However, the control of zeolite framework composition is limited, precluding its use in processes that require different acidic and ion-exchange properties. Here, JZO zeolites with tunable framework Si/Al ratios of *ca.* 15, 30, and 50 were successfully synthesized through precise adjustment of synthesis parameters, thereby broadening the synthesis window of this intriguing zeolite. A series of JZO zeolites was synthesized *via* a combination of the seed-assisted and the “deficient fluoride” methods. The crystallization kinetics of JZO was prominently boosted compared to the seed-free and fluoride-free counterpart systems, thanks to the accelerated formation of primary building units of JZO. Comprehensive characterization methods were employed to unveil the concerted role of seeds and fluoride ions. Dimethoxymethane carbonylation to methyl methoxyacetate reaction, a promising alternative route for producing degradable plastic monomers, was evaluated over JZO zeolites, and high activity (*ca.* 58%) and selectivity (*ca.* 84%) were achieved in JZO zeolite with Si/Al = 30 obtained by seed-assisted approach over a time-on-stream (TOS) test of 50 h. The product distribution could be rationally controlled by adjusting zeolite compositions. This work expands the viability of JZO zeolite synthesis and lays the foundation for practical applications.

## Abstract 2

Extra-large-pore JZO zeolite with a tunable Si/Al ratio demonstrated promising catalytic performance in dimethoxymethane carbonylation.

## Graphical abstract

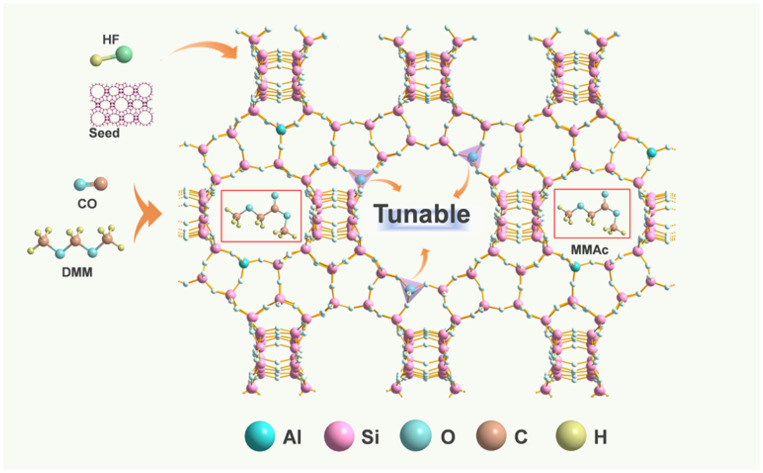

## Introduction

Zeolites represent a pivotal class of inorganic microporous materials. Endowed with unique porous structures, large specific surface areas, tunable active sites, and excellent thermal/hydrothermal stability, they have been extensively utilized in diverse fields, particularly in heterogeneous catalysis, ion exchange, and adsorption processes.^1,2^ A fundamental challenge in heterogeneous catalysis lies in the synthesis of hydrothermally stable zeolites possessing both extra-large pores (>0.75 nm) and strong acid sites.^3,4^ Extra-large-pore zeolites are distinguished by their highly accessible large pores and intrinsic acidity, which are advantageous not only for the processing of bulky reactants but also for mitigating diffusion limitations and improving the accessibility of acid sites in catalytic reactions.^5,6^ These advantageous properties render extra-large-pore zeolites particularly attractive for industrial applications.^7,8^ In 2021, Chen *et al.* reported a hydrothermally stable aluminosilicate zeolite, designated as ZEO-1 (IZA code: JZO), which represents the first documented example of extra-large pore zeolites containing 16-membered ring openings and a three-dimensional interpenetrating pore architecture.^9^ As an aluminosilicate zeolite, JZO inherently possesses framework-confined acidic active sites. In heavy oil catalytic cracking, it exhibits a conversion efficiency superior to that of commercial zeolite catalysts (*e.g.*, MFI, and Beta), and comparable to that of top-performing USY catalysts. JZO and its carbon-derived material have demonstrated remarkable performance in numerous catalytic reactions.^10^ JZO zeolite was successfully applied as a catalyst in the carbonylation and disproportionation reactions of dimethoxymethane, where its large reaction spaces and spatially distributed acid sites were reported to promote molecular diffusion and catalytic performance compared with conventional zeolites.^11^ Yu *et al.* successfully confined CsPbBr_3_ nanocrystals within JZO zeolite, achieving highly efficient photocatalytic hydrogen production in aqueous environments and highlighting its potential for broad application in sustainable photocatalytic processes.^7^ Bieseki *et al.* successfully synthesized a novel zeolite-templated carbon (ZTC) using the extra-large pore zeolite JZO as a template.^12^ The resulting material exhibited exceptional electrical conductivity and remarkable electrochemical performance, demonstrating significant potential for advanced energy storage applications. The remarkable performance of this novel extra-large-pore zeolite in catalytic applications underscores its significant potential for both academic study and industrial use.

Zeolites are crystalline aluminosilicates constructed from corner-sharing [SiO_4_]^4−^ and [AlO_4_]^5−^ tetrahedra.^13,14^ The substitution of Si^4+^ by Al^3+^ within the framework introduces a net negative charge, which is balanced by extra-framework cations. These sites constitute the acidic centers that govern the ion-exchange, catalytic, and adsorption properties of zeolite materials.^15,16^ This critical parameter directly modulates key characteristics of zeolites, including acidity, thermal stability, hydrothermal resistance, pore architecture, and surface properties.^17^ For instance, the surface functionality of a zeolite is dominated by hydroxyl groups (–OH) and Brønsted, whose density and strength can be precisely tailored by varying the framework Si/Al ratio.^18^ These functional groups serve as pivotal adsorption sites, playing a crucial role in the capture and removal of volatile organic compounds (VOCs) as well as catalytic applications. Furthermore, the Si/Al ratio governs the hydrophobicity/hydrophilicity of zeolites,^19^ which significantly influences its performance in solvent dehydration and recovery processes.^20–22^ In general, zeolites with lower Si/Al ratios exhibit higher cation-exchange capacity and Brønsted acid density, which enhance ion-exchange capacity and catalytic performance in certain reactions. Conversely, high Si/Al ratio zeolites (*e.g.*, ZSM-5 and Silicalite-1) are characterized by stronger hydrophobicity and improved anti-coking properties, and have demonstrated excellent performance in reactions such as methanol-to-olefins (MTO).^23,24^ Moreover, ZSM-5 zeolites with hierarchical pore architectures have been observed to exhibit distinct catalytic behaviors in the MTO reaction. Specifically, a ZSM-5 zeolite catalyst featuring extrinsic mesopores and intrinsic micropores can reduce coke formation rates and enhance diffusion efficiency, thereby improving catalytic stability and overall performance.^25^ Building on the understanding of structure–property relationships, JZO zeolite represents a unique case due to its intrinsic micropores and extra-large pores, which provide enlarged reaction space and reduce steric constraints, while the spatial distribution of framework aluminum enables more isolated and accessible acid sites, thereby creating a favorable environment for stabilizing reaction intermediates and regulating reaction pathways. However, according to previous reports, JZO zeolite typically has a framework Si/Al ratio of around 25, which limits the modulation of its hydrothermal stability, acid strength, and hydrophobicity, thereby constraining its performance in catalytic and adsorption applications. Thus, precise control of the Si/Al ratio in JZO zeolite, coupled with optimization of its synthesis strategy, is critical for developing high-performance, versatile zeolite materials.

Conventional approaches for modulating the zeolite compositions typically rely on the adjustment of the proportions of silica and alumina sources, mineralizers and structure-directing agents.^26,27^ The synthesis of high-silica or high-alumina zeolites, however, frequently encounters challenges such as low crystallinity, the formation of impurity phases, and extended crystallization durations. Accordingly, seed-assisted synthesis has emerged as a prominent strategy, owing to its efficacy in accelerating crystallization kinetics, improving phase purity, and precisely regulating crystal morphology.^27–29^ The crystallization-promoting mechanism of the seed primarily involves three aspects: (1) providing heterogeneous surfaces for epitaxial growth and reducing the activation energy required for nucleation; (2) directing the anisotropic growth of specific crystallographic planes to regulate the ultimate crystal morphology; (3) suppressing the formation of thermodynamically competitive impurity phases, thereby enhancing the selectivity toward the target zeolite. Taking the synthesis of high-silica ZSM-5 as an example, the introduction of nano-size seeds not only significantly accelerates crystallization kinetics but also reduces organic template usage,^30,31^ thereby lowering production costs.

In this study, the synthesis of JZO zeolites with different Si/Al ratios was explored using a combined strategy of the “deficient fluoride” method^32^ and a seed-assisted approach. With the employment of nano-seeds, hydrofluoric acid addition and water content control in the synthesis gel, phase-pure JZO zeolites with various framework Si/Al ratios of *ca.* 15, 30, and 50 were successfully synthesized with accelerated crystallization kinetics. The physicochemical properties of JZO zeolites were elucidated *via* multiple characterization techniques, including PXRD, XRF, SEM, TEM, N_2_ physical adsorption, solid-state NMR, NH_3_-TPD, and Py-FTIR. The role of seeds in the synthesis of JZO was investigated by analyzing products extracted at different stages of crystallization. Finally, the catalytic performance of JZO zeolites in the carbonylation of dimethoxymethane to methyl methoxyacetate, an alternative route for degradable plastic monomer production, was evaluated. Although DMM is not a particularly bulky molecule, previous studies have shown that this reaction is strongly influenced by pore architecture, diffusion behavior, and the spatial distribution of acid sites. Therefore, the dependence of catalytic performance on the Si/Al ratio and aluminum distribution in JZO was discussed.

## Experimental

### Materials

All chemicals were used as received without further purification: tricyclohexylphosphine (Energy Chemical, 98%), iodomethane (Energy Chemical, 99.5%), aluminum isopropoxide (Al(O-iPr)_3_, Shanghai Mcklin, AR), hydrofluoric acid (HF, European Pharmacopoeia, 48%), tetraethyl orthosilicate (TEOS, Sinopharm, AR); acetonitrile (Sinopharm, AR), ethanol (Sinopharm, AR), acetone (C_3_H_6_O, Sinopharm, AR), ammonium chloride (Sinopharm, AR), anion exchange resin (Xidian Technology, 1.1 mequiv. per mL),standard hydrochloric acid (Metrological Group, 0.1003 M), CO (Dalian Special Gas Corporation, 99.99%) and dimethoxymethane (DMM, Aladdin, 98%).

### OSDA synthesis

The organic structure-directing agent (OSDA) was synthesized according to a previously reported procedure.^9^ Typically, 85.86 g (0.3 mol) of tricyclohexylphosphine was added to a round-bottom flask containing 750 mL of acetonitrile. The flask was placed in an ice bath under continuous magnetic stirring. Then, 149.79 g (1.05 mol, 3.5 e.q.) of iodomethane was added dropwise *via* a constant-pressure dropping funnel. After the reaction was allowed to proceed overnight at room temperature, the mixture was rotary-evaporated under reduced pressure to remove acetonitrile and unreacted iodomethane, yielding a white solid powder identified as tricyclohexylmethylphosphonium iodide (yield: 96%). The purity and structure of the OSDA were confirmed by ^1^H and ^13^C NMR spectroscopy (TCyMPOH, Fig. S1) in deuterated chloroform. The iodide form was subsequently converted to the corresponding hydroxide form (TCyMPOH) using an anion exchange resin. The resulting hydroxide solution was concentrated using rotary evaporation and its concentration was determined by titration with 0.1 M HCl.

### Zeolite synthesis

The typical seed-free synthesis of JZO zeolite employs HF as the fluorine source, following our previously reported ‘fluoride-deficient’ method,^32^ where stoichiometrically deficient fluoride anions to that of OSDA was added. The molar composition of the synthesis gel is 1.0 SiO_2_: *x*Al_2_O_3_: 0.5 TCyMPOH: 0.2 HF: 5H_2_O. A detailed procedure for the synthesis with a target Si/Al ratio of 15 (*x* = 0.033) is as follows: 0.278 g (1.33 mmol) Al(O-iPr)_3_ was dissolved in 12.302 g (10 mmol) of TCyMPOH solution in a plastic beaker under stirring at 450 rpm for 20 min to form a transparent sol. 4.167 g (20 mmol) TEOS was then added, and the mixture was hydrolyzed overnight at room temperature. The transparent sol was transferred to an oven and aged at 100 °C to evaporate excess water and ethanol until a predefined weight was reached. Under a fume hood, 0.145 mL HF (4 mmol) was added *via* micropipette, followed by manual stirring for 15 min to achieve homogeneity. The gel was transferred into a PTFE-lined autoclave and then heated at 190 °C for 9 days in a convection oven. After cooling to room temperature, the product was centrifuged at 15 000 rpm and sequentially washed with 40 mL deionized water, 20 mL anhydrous ethanol, and 10 mL acetone. The wet powdery product was dried in a convection oven at 100 °C for 2 h. For a typical seed-assisted synthesis using a gel molar composition of 1.0 SiO_2_: 0.033 Al_2_O_3_: 0.5 TCyMPOH: 0.2 HF: 5H_2_O, JZO seeds (0.028 g, 5 wt% relative to silica) were introduced into the prepared gel and mixed thoroughly until a uniform viscous gel was obtained. The crystallization procedures were the same as the above seed-free method. The final as-made products were denoted as *xx*-s (5 wt% seed)/n (no seed)-as. For example, 15 s-as represents a sample synthesized with 5 wt% seeds and a Si/Al ratio of 15.

The OSDA was removed from the as-made JZO *via* calcination according to a reported procedure.^10^ The calcination was performed in a muffle furnace under static air using the following temperature program: ramping from room temperature to 150 °C in 45 min, holding at 150 °C for 60 min to remove physisorbed water and volatile species, heating to 600 °C over 225 min, and maintaining at 600 °C for 6 h to completely combust the OSDA. Samples treated in this way are denoted as *xx*-s (5 wt% seed)/n (no seed)-cal, where “*xx*” indicates the nominal Si/Al ratio. For instance, 15 s-cal refers to a calcined sample synthesized with 5 wt% seed and a target Si/Al ratio of 15. To eliminate residual phosphorus species, 1 g of the calcined JZO was transferred into a sealed 50 mL plastic bottle, mixed with 20 mL of 0.5 M ammonium chloride solution, and stirred at 400 rpm for 3 h at 60 °C in an oil bath. The solid was recovered by centrifugation at 15 000 rpm for 5 min, washed repeatedly with deionized water until the supernatant reached near neutral pH, and the ion-exchange process was repeated twice. The product was dried overnight at 100 °C. These samples are labelled as *xx*-s (5 wt% seed)/n (no seed)-cnw (*e.g.*, 15 s-cnw: with a gel Si/Al ratio of 15, 5 wt% seeded, calcined, and ammonium-washed).

### Characterizations

Powder X-ray diffraction (XRD) patterns were acquired using a Rigaku SmartLab diffractometer equipped with Cu Kα radiation (*λ* = 1.5406 Å). Measurements were performed in Bragg–Brentano (*θ*–*θ*) geometry over the 2*θ* range of 2–50° with a step size of 0.01°. Scanning electron microscopy (SEM) images were collected with a Hitachi S-4800 microscope equipped with a cold-field emission gun, operated at 2 kV. High-resolution transmission electron microscopy (HRTEM) images were acquired on a JEOL-3200FS field-emission transmission electron microscope at an accelerating voltage of 300 kV, and images were recorded using a Gatan OneView CMOS camera. Thermogravimetric and differential thermal analysis (TG-DTA) was performed using a Rigaku Thermo Plus EVO II TG-DTA8122 instrument. Measurements were conducted under a flowing air atmosphere (50 mL min^−1^) with a heating rate of 10 °C min^−1^ from 30 to 1000 °C. The silicon and aluminum content in the samples was primarily determined by X-ray fluorescence spectroscopy (XRF, ZSX Primus IV, Rigaku) operated in SQX mode for automated quantitative analysis. The results were further verified by inductively coupled plasma optical emission spectrometry (ICP-OES, PerkinElmer Nexion 350X) after sample digestion *via* alkali fusion. Nitrogen physisorption isotherms were measured at 77 K using a Quantachrome Autosorb IQ analyzer. The sample was degassed under vacuum at 300 °C for 6 h to remove physisorbed contaminants prior to analysis. Ammonia temperature-programmed desorption (NH_3_-TPD) measurements were performed using a fixed-bed reactor equipped with a thermal conductivity detector (TCD). Prior to the analysis, the sample was pretreated at 100 °C for 1 h under flowing N_2_ to remove physisorbed species. The sample was then saturated with a mixture of NH_3_/N_2_ for 30 min at 100 °C. Subsequently, physically adsorbed ammonia was removed by purging with N_2_ for 10 min. The temperature was then increased from 100 to 800 °C at a constant heating rate, and the NH_3_ desorption profile was recorded to evaluate the total acidity and acid strength distribution. Pyridine adsorption Fourier transform infrared (Py-FTIR) spectroscopy was conducted on a Thermo Scientific Nicolet iS50 FTIR spectrometer at a spectral resolution of 4 cm^−1^. The sample was pressed into a self-supporting pellet (diameter: 1.6 cm, mass: 20 mg) and placed in a custom-designed infrared cell. The pellet was activated under vacuum at 450 °C for 4 h, cooled to 25 °C, and a background spectrum was recorded. Pyridine vapor (equilibrium pressure: 1.2 Torr) was then introduced at 100 °C for 10 min. After subsequent degassing at 200 °C to remove physisorbed pyridine, the spectrum was recorded at 25 °C. All solid-state NMR spectra were recorded on a Bruker 500NB Avance III spectrometer operating at 11.7 T (*ν*_0_(^1^H) = 500 MHz). ^27^Al single pulse MAS NMR spectra were recorded using 3.2 mm rotors spun at *ν*_MAS_ = 14 kHz. A pulse duration of 1.75 µs corresponding to a flip angle of π/12 and a recycling delay of 1 s was employed. Chemical shifts were referenced to AlCl_3_.

### Performance evaluation in dimethoxymethane carbonylation

DMM carbonylation was carried out in a fixed-bed reactor. The reactor was equipped with a quartz-lined stainless-steel tube (inner diameter: 8 mm), and the reaction temperature was monitored by a K-type thermocouple. The system pressure was controlled by a back-pressure valve. The catalyst (0.05–0.10 g) was loaded in the center of the reactor and supported by quartz wool. Prior to reaction, the catalyst was pretreated in a flow of N_2_ at 773 K for 1 h. Then, DMM mixed with CO was introduced into the reactor at 363–383 K and 0.5–2 MPa, with a CO flow rate of 30 mL min^−1^, *n*_CO_/*n*_DMM_ ratios of 57–114, and WHSV_DMM_ of 0.98–1.98 h^−1^. WHSV_DMM_ was defined as the mass flow rate of DMM per unit mass of catalyst. The products were analyzed online using a gas chromatograph (Agilent 7890B) equipped with an HP-PLOT-Q column and a flame ionization detector (FID). DMM conversion and product selectivities were calculated based on carbon balance. A simplified reaction scheme for DMM conversion is shown below, illustrating the main carbonylation pathway to methyl methoxyacetate (MMAc) and the competing disproportionation pathway to methyl formate (MF) and dimethyl ether (DME). More detailed reaction mechanisms have been reported in the literature.^11^

Main reaction:DMM + CO → MMAcCH_3_OCH_2_OCH_3_ + CO → CH_3_OCH_2_COOCH_3_

Competing side reaction (disproportionation):DMM → MF + DMECH_3_OCH_2_OCH_3_ → HCOOCH_3_ + CH_3_OCH_3_

## Results and discussion

### Zeolite synthesis

The synthesis conditions and products obtained are summarized in Table 1. The syntheses were performed at 190 °C using a gel molar composition of 1.0 SiO_2_: *x*Al_2_O_3_: 0.5 TCyMPOH: 0.2 HF: *y*H_2_O, with and without seeds (5 wt%). Well-crystalline JZO was obtained within 5 days with Si/Al ratios of 15–50 in the gel (Fig. S2 and Table 1, Entries 10–20). However, when the Si/Al ratio was further increased to 70, the XRD patterns show the characteristic diffraction peaks of JZO, indicating that JZO is the dominant crystalline phase. Meanwhile, SEM images reveal the presence of a small amount of plate-like phase (Fig. S3), which is considered an unknown precursor as reported previously,^32^ suggesting the coexistence of a minor impurity phase. Elemental analysis of this mixture phase shows a Si/Al ratio of *ca.* 50 (Table S1), suggesting limited aluminum incorporation under these conditions. As the gel Si/Al ratio further increased to 80 and 100, JZO with amorphous phase and solely amorphous phase were detected, respectively, under the crystallization time investigated (Table 1, Entries 27–30). The same trend was also observed with further decrease of Si/Al from 15 to 5 (Table 1, Entries 1–8). Additionally, water content was found to be a vital factor in modulating crystallization kinetics. At a higher water content (*y* = 10, H_2_O/SiO_2_ = 10), amorphous or poor-crystallized JZO was obtained at similar heating times (Fig. S4 and Table 1, Entries 2, 5, 9, 12, and 18). In contrast, well-crystalline JZO was consistently obtained at the lower water content (*y* = 5, H_2_O/SiO_2_ = 5) within shorter heating times of 5 days. These results clearly demonstrate that a lower H_2_O/SiO_2_ ratio of 5 (*y* = 5) is more conducive to the synthesis of phase-pure JZO, enabling the consistent preparation of highly crystalline products. The enhanced crystallization efficiency under reduced water content could be attributed to changes in gel chemistry and enhanced interaction between inorganic and organic species. The higher local concentration of OSDA and the increased viscosity promote more effective mass transfer between the silicon and aluminum precursors and OSDA.^33^ This optimized environment accelerates nucleation and directs the growth toward the formation of JZO. At higher H_2_O content (*y* = 10), the excessive dilution of the gel system weakens the interaction between the OSDA and aluminosilicate species. As a result, nucleation is hampered, crystallization kinetics are slowed, and the formation of amorphous aluminosilicate phases or mixed zeolitic by-products is favored.^34^

**Table 1:** Summary of synthesis conditions and phases obtained with different Si/Al ratios using HF

Entry	Si/Al	H_2_O/SiO_2_	Time (day)	Seed	Phase^a^
1	5	5	5	Yes	Am.
2	7.5	10	8/11	Yes	Am.
3		5	5/8		Am. (JZO)
4			11		JZO (Am.)
5	10	10	8/11	Yes	Am.
6		5	5/8/11		Am. (JZO)
7	12.5	5	5	Yes	Am. (JZO)
8			8		JZO (Am.)
9	15	10	5/8	Yes	Am.
10		5	5/7/9		JZO
11	15	5	5/7/9	No	JZO
12	30	10	5	Yes	Am. (JZO)
13		5	5/7/9		JZO
14	30	5	5/7/9	No	JZO
15	40	5	5/7	Yes	JZO
16			9		JZO (Am.)
17	40	5	5/7/9	No	JZO
18	50	10	5	Yes	JZO (Am.)
19		5	5/7/9		JZO
20	50	5	5/7/9	No	JZO
21	60	5	5/9	Yes	JZO (Am.)
22			7		JZO
23	60	5	5/7	No	JZO (Am.)
24			9		JZO
25	70	5	5/7/9/11	Yes	JZO (unknown)
26			5/7/9/11	No	JZO (unknown)
27	80	5	5/7/9	Yes	JZO (Am.)
28		5	5/7/9	No	JZO (Am.)
29	100	5	5/7/9/11	Yes	JZO (Am.)
30			5/7/9/11	No	Am.

a Am. denotes amorphous phase; the minor phases were given in parentheses.

To address the toxicity, high corrosivity, and handling difficulties associated with HF in industrial applications, we investigated the feasibility of synthesizing JZO zeolites without HF. The products obtained under various synthesis conditions are summarized in Table S2. The results demonstrate that, with the assistance of seeds, JZO zeolite could be synthesized across a broad gel Si/Al ratio range of 10–100 (Fig. S5). Nevertheless, this HF-free approach presents notable limitations, including low product yield, limited aluminum incorporation, and poor reproducibility. These observations can be interpreted in terms of seeding and the constraints of the HF-free system: the introduction of 5 wt% JZO seed fragments provides predetermined nucleation sites, enabling heterogeneous crystallization of silicon and aluminum species and bypassing the homogeneous nucleation barrier inherent in low-activity, acid-free environments.^35^ Over a wide range of gel Si/Al ratios (10–100), the seeds direct the assembly of aluminosilicate precursors according to their inherent framework topology, resulting in phase-pure JZO. However, fluoride ions are essential for activating the cleavage of Si–O bonds. In the absence of fluoride ions, the dissolution and depolymerization of silica sources are markedly suppressed.^36^ While HF promotes the transformation of SiO_2_ into highly reactive fluorosilicate intermediates (*e.g.*, [SiO_2_F]^−^), the HF-free system leaves silicon predominantly in low-activity polymeric states such as oligomeric silicic acids.^37^ These species are unable to rapidly convert into the monomeric or low-oligomeric silicates necessary for zeolite framework assembly. As a result, a substantial fraction of the silicon and aluminum species does not integrate into the crystalline product, instead remaining in the mother liquor as amorphous precipitates or soluble oligomers. This leads to significantly reduced product yield (calculated as the mass of crystalline powder product ((as-made)/gel mass). Furthermore, as can be seen from the XRF results and calculated yields in Table S2, the mismatch in reactivity between aluminum and silicon precursors is exacerbated in the absence of HF, since the aluminum species are less effectively incorporated into the growing framework.^38,39^ This results in pronounced deviations in Si/Al ratios in the gel and in the final crystalline product. Together, these results underscore the critical role of HF in regulating precursor reactivity, facilitating Al insertion, and enabling the efficient synthesis of JZO zeolites with well-controlled Si/Al ratios.

### Structural characterizations

**Fig. 1:**
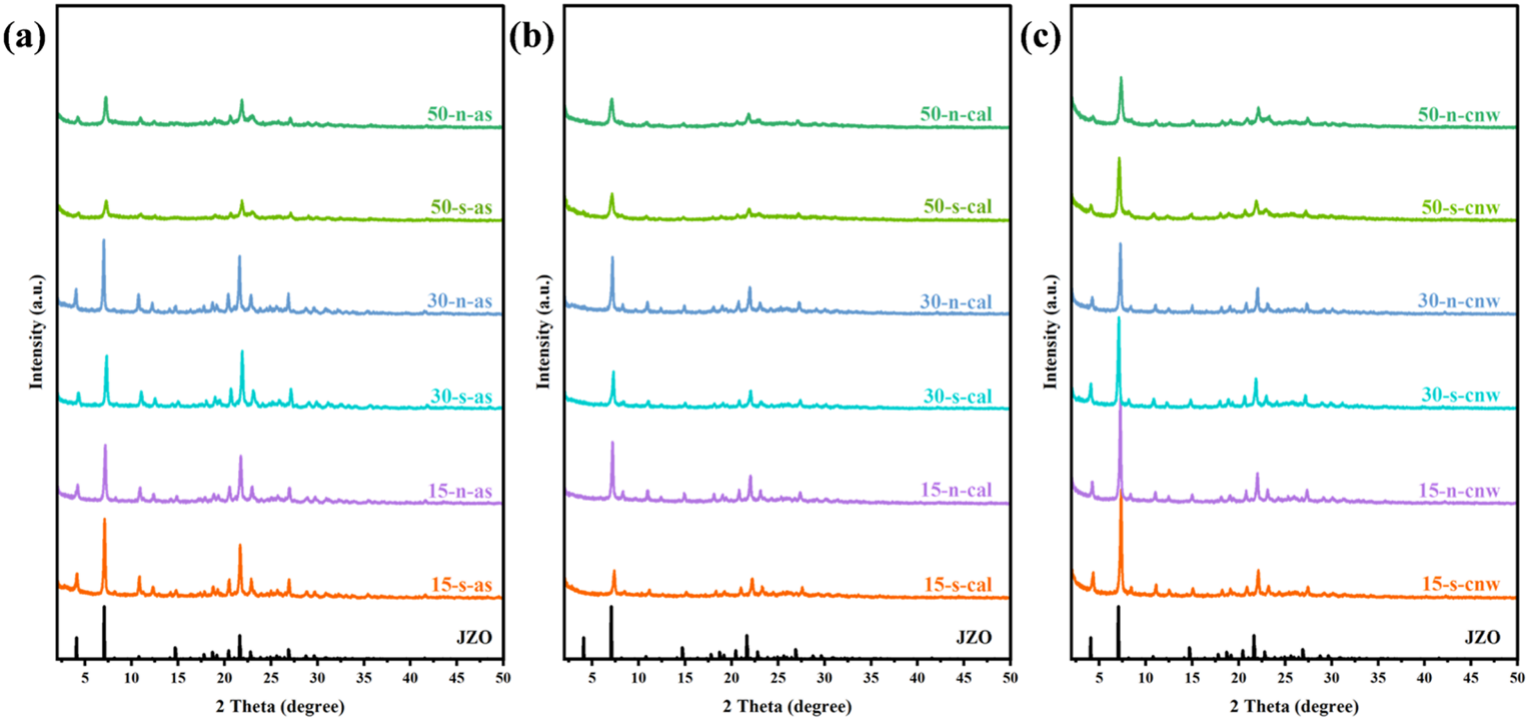
PXRD patterns of as-made (a), calcined (b), and subsequently NH_4_Cl-washed (c) JZO zeolites obtained with different Si/Al ratios. The gel molar composition is 1.0 SiO_2_: 0.5 TCyMPOH: *x*Al_2_O_3_: 0.2 HF: 5H_2_O, with *x* = 0.010, 0.017, 0.033 corresponding to Si/Al ratios of 50, 30, 15, respectively. s-5 wt% seed, n-no seed.

Fig. 1 displays the PXRD patterns of JZO zeolites synthesized with a F/SiO_2_ ratio of 0.2 and the gel Si/Al ratios of 15, 30, and 50, in their as-made (as), calcined (cal), and subsequent NH_4_Cl washing (cnw) states. All samples display characteristic diffraction peaks that closely match the simulated XRD pattern of JZO. No impurity phases were detected, confirming the successful synthesis of pure and well-crystalline JZO across all compositions. A noticeable decrease in crystallinity was observed after calcination compared to the as-made samples. However, the subsequent ammonium washing not only removed residual phosphorus from the samples but also restored and even enhanced the crystallinity relative to the parent material. This is because the ammonium washing removes residual phosphorus and other guest species that remain in the channels after calcination, thereby increasing the relative contribution of the crystalline phase to the diffraction signal. Although all samples maintain phase purity, variations in peak intensity, width, and resolution are observed with varying Si/Al ratios, suggesting possible variations in crystallite size or crystallinity. A detailed discussion of these effects will be provided in subsequent sections. The PXRD patterns of samples obtained with different Si/Al ratios at different crystallization times are presented in Fig. S6. The results show that for zeolites synthesized with different Si/Al ratios, if the PXRD patterns of the samples show impure phases within the initial 5 or 7 days, extending the crystallization time does not yield phase-pure JZO zeolites. Moreover, prolonging the time weakens the PXRD pattern intensities, indicating crystal dissolution, and reduced crystallinity.

**Fig. 2:**
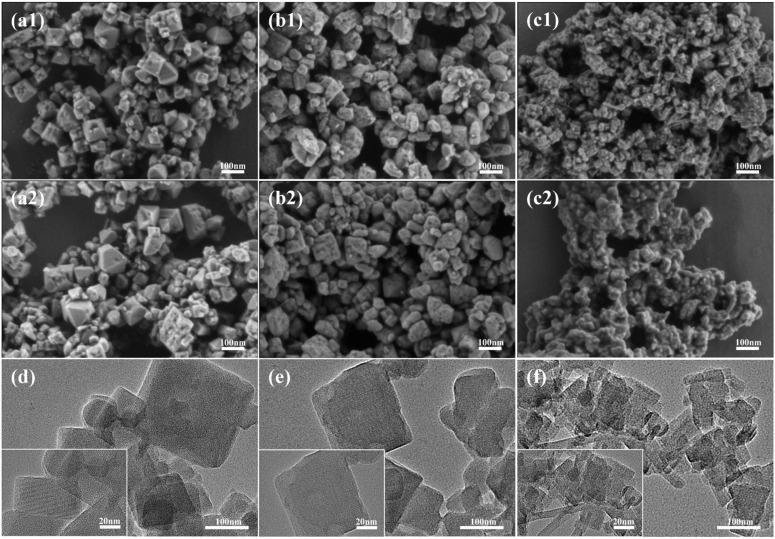
SEM (a–c) and TEM (d–f) images of as-made JZO zeolites obtained with different Si/Al ratios: 15 s-as (a1 and d), 15-n-as (a2), 30 s-as (b1 and e), 30-n-as (b2), 50 s-as (c1 and f), and 50-n-as (c2). The gel molar composition is 1.0 SiO_2_: 0.5 TCyMPOH: *x*Al_2_O_3_: 0.2 HF: 5H_2_O, with *x* = 0.010, 0.017, 0.033 corresponding to Si/Al ratios of 50, 30, 15, respectively. s-5 wt% seed, n-no seed.

SEM and TEM images of as-made JZO with Si/Al ratios of 15, 30, and 50 are presented in Fig. 2. The samples with a Si/Al ratio of 15 (Fig. 2a1 and a2) consist of nanocrystals with well-defined truncated octahedral morphology, relatively narrow particle size distribution (30–100 nm), and smooth surfaces. The particles exhibit a relatively uniform morphology and size distribution. For the sample with a Si/Al ratio of 30 (Fig. 2b1 and b2), crystals exhibit irregular polyhedral shapes with apparent fractures and eroded edges, together with some particles that possess recognizable cubic or octahedral outlines. The crystallite size ranges from 20 to 100 nm, with finer fragments likely originating from broken larger crystals. In contrast, the Si/Al ratio of 50 sample (Fig. 2c1 and c2) exhibits a distinctly different morphology, characterized by aggregates of nanoplates and irregular particles with sizes of around 20–50 nm. The high-resolution TEM images of JZO zeolites with the three different Si/Al ratios reveal well-shaped crystals with continuous lattice fringes and regular lattice spacings, while amorphous phases or impurity particles are not detected (Fig. 2d–f), corroborating the high crystallinity of the obtained JZO zeolites. Notably, SEM images reveal that the sample with a Si/Al ratio of 70 (Fig. S3a and b) exhibits two different morphologies, including nanoparticles and nanoplates. The latter could be ascribed to an unknown precursor phase or to a pure silica phase, and warrants a dedicated study in the future. The former nanoparticles, in contrast, is ascribed to JZO zeolite crystals with particle sizes range of 20 to 50 nm. These morphological variations correlate well with changes in peak width, intensity, and resolution in the corresponding PXRD patterns (Fig. 1), confirming that the initial Si/Al ratio is a key factor governing target phase, crystal size, and morphology.

### Composition analysis of JZO zeolites

Elemental composition (XRF, ICP) and thermal gravimetric (TG) analysis were carried out on JZO samples prepared with an F/SiO_2_ ratio of 0.2, H_2_O/SiO_2_ ratio of 5, and varying nominal Si/Al ratios to determine the actual Si/Al ratios and the status of the OSDA in the as-made zeolites (Table 2). For the ammonium-washed samples (denoted as cnw), the Si/Al ratios are close to the nominal ratios in the starting gel despite minor discrepancies, confirming the successful incorporation of most of the aluminum into JZO. In addition, ICP analysis detected only a trace amount of phosphorus (0.05 wt%) in the 30 s-cnw sample, indicating that only a small amount of residual P species remained after the ammonium washing treatment. Thermogravimetric analysis was conducted at a temperature range from 25 to 1000 °C, and all samples exhibited similar weight loss behavior (Fig. S7a). The weight loss below 200 °C was attributed to the removal of physisorbed water. Notably, the weight loss below 200 °C increased with rising Si/Al ratio, from 1.73 wt% to 3.41 wt%, indicating slightly enhanced hydrophilicity in higher-silica JZO zeolites, which is contradictory to the increase hydrophobicity trend in high-silica zeolite and might be explained by the existence of structural defects. A major mass loss occurred between 200 and 600 °C, corresponding to the combustion and removal of the OSDA from the zeolite pores. Moreover, the weight loss in the 400–600 °C range increased with increasing Si/Al ratio, indicating that a larger amount of OSDA species was retained within the zeolite products. This trend is consistent with previous reports showing that OSDA incorporation can increase with increasing Si/Al ratio in zeolites.^40^ The corresponding differential thermal analysis (DTA) curves showed an endothermic peak between 25 and 100 °C, a weak exothermic peak at 300–400 °C, a strong exothermic peak at 350–450 °C, and a broad weak exothermic signal around 600 °C (Fig. S7b). Based on the OSDA decomposition mechanism, these features can be assigned as follows: the weak exotherm (300–400 °C) corresponds to the Hoffmann degradation of TCyMP^+^ cations; the strong exotherm (350–450 °C) arises from the combustion of TCyMP^+^ and charge compensation for framework aluminum; the broad exotherm near 600 °C is associated with oxidation of residual carbonaceous species.^32,41^

**Table 2:** Elemental analysis and composition of JZO zeolites obtained with different Si/Al ratios using HF

Zeolite	XRF			ICP				Zeolite	TG weight loss (%)	
	SiO_2_%	Al_2_O_3_%	Si/Al	P%	Si%	Al%	Si/Al		<200 °C	200–1000 °C
15-s-cnw	94.50	4.79	16.77	—	25.78	1.76	14.14	15-s-as	1.73	20.79
15-n-cnw	34.90	4.50	17.93	—	—	—	—	15-n-as	2.17	19.50
30-s-cnw	96.92	2.80	29.45	0.05	28.62	1.03	27.79	30-s-as	2.80	21.02
30-n-cnw	96.97	2.78	29.65	—	—	—	—	30-n-as	2.20	22.01
50-s-cnw	98.02	1.77	47.20	—	32.51	0.63	49.50	50-s-as	3.41	25.70
50-n-cnw	97.96	1.82	45.86	—	—	—	—	50-n-as	3.37	23.71

### Porosity analysis

**Fig. 3:**
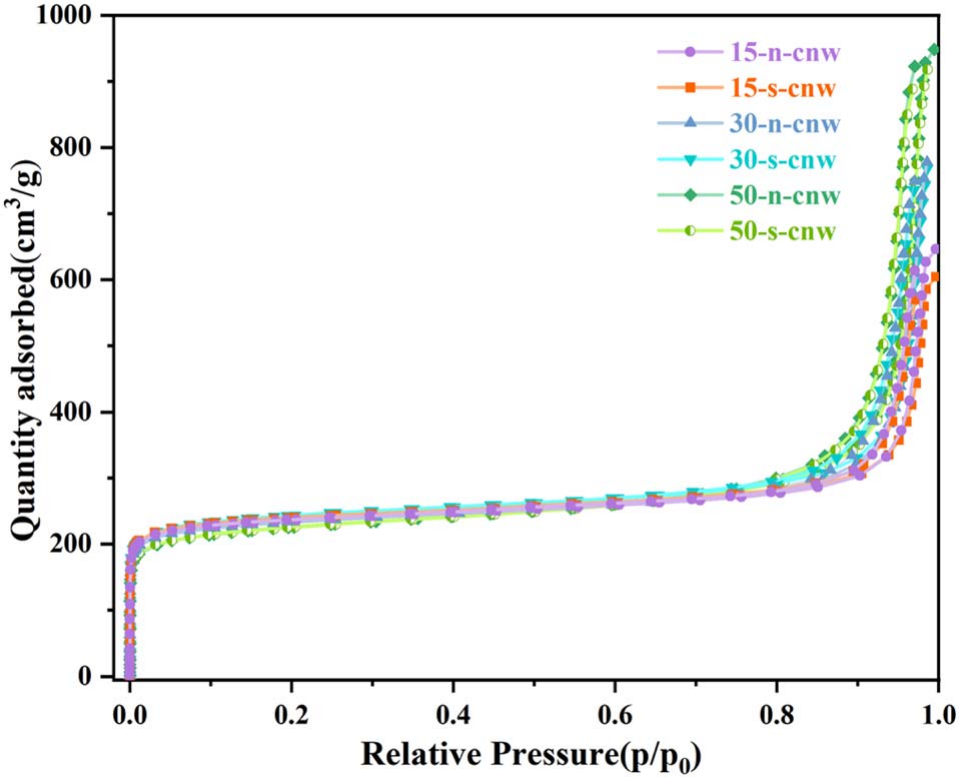
N_2_ adsorption–desorption isotherms of cnw-JZO zeolites with different Si/Al ratios. The gel molar composition is 1.0 SiO_2_: 0.5 TCyMPOH: *x*Al_2_O_3_: 0.2 HF: 5H_2_O, with *x* = 0.010, 0.017, 0.033 corresponding to Si/Al ratios of 50, 30, 15, respectively. s-5 wt% seed, n-no seed.

To investigate the textural properties of JZO zeolites with different Si/Al ratios, N_2_@77K physisorption analysis over the ammonium-washed (cnw) JZO samples was performed (Fig. 3). As shown in Fig. 3, all samples exhibit Type I isotherms with comparable micropore volumes. A sharp uptake step is observed at *P*/*P*_0_ > 0.8, indicating the presence of mesopores formed by interparticle stacking or crystal aggregation. The pore volume was calculated using the t-plot method, and the specific surface area was determined by the Rouquerol BET criteria (Table S3), both of which serve as crucial indicators of crystallinity. In contrast to PXRD, which primarily reflects the long-range crystalline order of the framework, nitrogen physisorption provides information on the accessible micropore volume.^42^ This offers complementary insight into the internal pore structure and supports the formation of well-developed and accessible pore systems in the obtained JZO zeolites.

Based on previous studies, well-crystallized JZO zeolites typically exhibit micropore volumes of 0.27–0.32 cm^3^ g^−1^, with an ideal value near 0.30 cm^3^ g^−1^.^32^ The samples synthesized in this work, with varying Si/Al ratios, all meet these benchmarks in specific surface area, characteristic pore size, and micropore volume, further confirming their high crystallinity and structural integrity.

### Acidity analysis and solid-state NMR

**Fig. 4:**
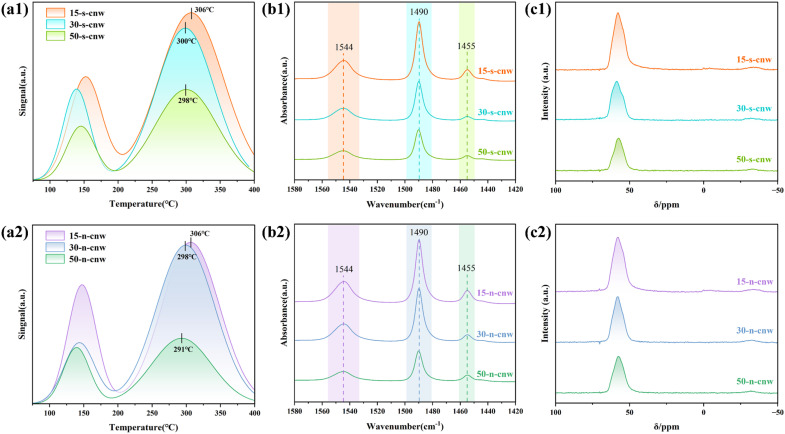
NH_3_-TPD curves (a1 and a2), pyridine adsorption FT-IR spectra (b1 and b2) and ^27^Al MAS NMR spectra (c1 and c2) of cnw-JZO obtained at different Si/Al ratios. The gel molar composition is 1.0 SiO_2_: 0.5 TCyMPOH: *x*Al_2_O_3_: 0.2 HF: 5H_2_O, with *x* = 0.010, 0.017, 0.033 corresponding to Si/Al ratio of 50, 30, 15, respectively. s-5 wt% seed, n-no seed.

The acidic properties of ammonium-washed JZO zeolites with different Si/Al ratios were evaluated using NH_3_-TPD, as shown in Fig. 4a1 and a2. The desorption profiles can be deconvoluted into two distinct regions: weak acid sites (100–200 °C), and medium-strength acid sites (250–400 °C). Quantitative fitting of the TPD curves (Table S4) reveals two clear trends with increasing Si/Al ratios: (1) the total acid site density decreases, as indicated by the reduction in total NH_3_ desorption area; (2) the high-temperature desorption peaks shift toward lower temperatures (from ∼306 to ∼298 °C in Fig. 4a1 and from ∼306 to ∼291 °C in Fig. 4a2), suggesting a weakening of medium-strong acid strength. These trends again reflect the successful modulation of the framework Si/Al ratios. As the aluminum content decreases, the number of [AlO_4_]^−^ tetrahedra and hence the number of protonic acid sites decreases, resulting in a decline in total acidity. The downward shift in high-temperature desorption further reflects the decreased strength of the remaining acid sites, attributed to reduced framework Al density and increased site isolation.^43^

Semi-quantitative analysis of Brønsted acid sites (BAS) and Lewis acid sites (LAS) in P-free JZO zeolites was carried out using pyridine adsorption infrared spectroscopy (Py-FTIR) (Fig. 4b1 and b2). Specifically, the band centered at *ca.* 1540 cm^−1^ is assigned to the ring vibration of protonated pyridine entities (PyH^+^) adsorbed at BAS, the band at *ca.* 1450 cm^−1^ corresponds to the vibrational mode of pyridine coordination entities adsorbed at LAS. The band at *ca.* 1490 cm^−1^ corresponds to the co-adsorption peak of pyridine at both BAS and LAS.^44,45^ The IR results indicate that the number of acid sites is significantly regulated by the Si/Al ratio (*i.e.*, aluminum content), with the peak areas at the three locations decreasing as the Si/Al ratios increase. Aluminum is recognized as the primary source of BAS (framework hydroxyls), such that a decreased Si/Al ratio (indicating increased aluminum content) leads to a marked enhancement in the intensity of the 1544 cm^−1^ peak, with the number of BAS subsequently increased. In contrast, an increased Si/Al ratio results in weakened intensity of this peak, and BAS abundance is reduced accordingly. Concurrently, the decreased Si/Al ratio (increased aluminum content) is typically accompanied by an increase in framework Al defects or non-framework Al species, which induces enhanced intensity of the 1455 cm^−1^ peak and a corresponding increase of LAS.

Furthermore, the acid site changes revealed by Py-FTIR can be further cross-validated by NH_3_-TPD results (Fig. 4a1 and a2) and ^27^Al NMR characterization (Fig. 4c1 and c2). The ^27^Al MAS NMR spectra of the cnw-JZO zeolites show a dominant resonance at *ca.* 59 ppm together with a shoulder at *ca.* 54 ppm, both characteristic of tetrahedrally coordinated framework aluminum species. A weak signal observed at around −30 ppm is attributed to a spinning sideband. No detectable signals corresponding to octahedrally coordinated extra-framework aluminum species are observed, indicating that aluminum is predominantly incorporated into the zeolite framework. A definitive assignment of Al to specific crystallographic T sites cannot be established based on one-dimensional ^27^Al MAS NMR alone.^46,47^ According to the reported structural model, JZO zeolite contains 21 crystallographic T sites.^10^ Among the samples, the JZO with a Si/Al ratio of 15 exhibits the highest signal intensity, consistent with its higher aluminum content.

### The role of seeds

**Fig. 5:**
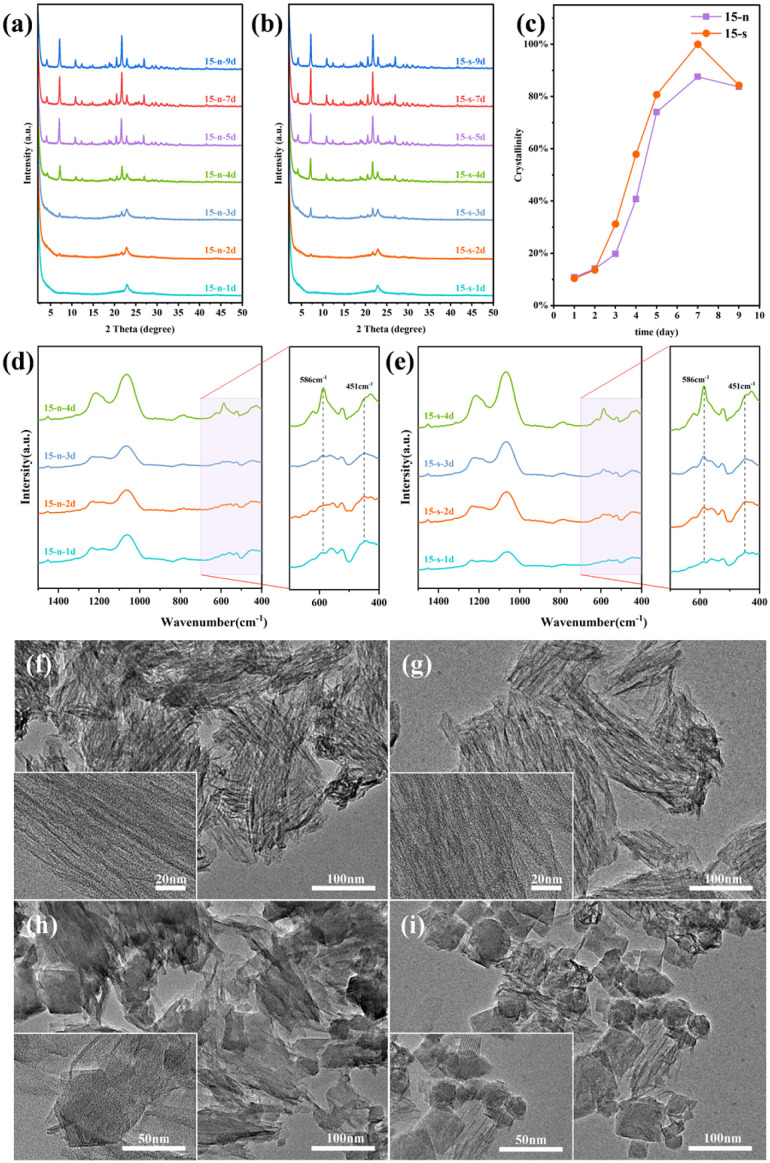
(a and b) XRD patterns of JZO samples synthesized without seeds (a) and with seeds (b). (c) Crystallization kinetics curves of JZO zeolite. (d and e) FTIR spectra of JZO synthesized without seeds (d) and with seeds (e). (f–i) TEM images of as-synthesized JZO samples: 15-n-1d (f), 15 s-1d (g), 15-n-3d (h), 15 s-3d (i). The gel molar composition of all the sample is 1.0 SiO_2_: 0.5 TCyMPOH: 0.033 Al_2_O_3_: 0.2 HF: 5H_2_O, Si/Al ratio of 15, s-5 wt% seed, n-no seed.

A systematic investigation into the role of seeding was conducted through comprehensive characterizations of the products obtained in different crystallization stages. The effect of seeds on the crystallization kinetics of zeolites synthesized with a gel Si/Al ratio of 15 was investigated over 1 to 9 days. PXRD patterns (Fig. 5a and b) and crystallization kinetic curves (Fig. 5c) reveal the evolution of the crystalline phases during synthesis. The relative crystallinity shown in Fig. 5c was calculated from the integrated peak areas of six characteristic diffraction peaks (2*θ* ≈ 4.05°, 7.04°, 10.78°, 20.5°, 21.7°, and 22.9°) of JZO, where peak areas were normalized to that of the corresponding peak areas in the reference sample (15 s-7d), whose crystallinity was defined as 100%. A similar precursor phase showed up after 1 day with a prominent XRD peak centered at *ca.* 23°. This precursor phase exhibits a wrinkled nanosheet morphology (Fig. 5f–g) and no JZO crystals could be observed, indicating the absence of JZO seeds in the seed-assisted system. After prolonged heating for 2 days, weak diffraction peaks assigned to JZO appeared alongside those of the precursor. The seeded system exhibited distinct characteristic diffraction peaks after 3 days of crystallization, and well-resolved diffraction peaks were observed after 4 days. In contrast, the characteristic diffraction peaks of the seed-free system were relatively weak after 3 days. After extending the crystallization time to 4 days, well-resolved characteristic diffraction peaks were also observed in the seed-free system, but their intensities remained lower than those of the seeded system. The TEM images show that the seeded system rendered numerous clusters of cubic nanocrystals with well-defined boundaries and uniform grain sizes, accompanied by lamellar precursor (Fig. 5i). In contrast, the seed-free sample still contains substantial lamellar precursor and irregular JZO crystals (Fig. 5h). The FTIR spectra of the fingerprint regions of the zeolite structure were collected for samples at crystallization times of 1 to 4 days (Fig. 5d and e). Absorption bands at *ca.* 450–550 cm^−1^ can be attributed to the bending vibration of O–Si–O bonds,^48,49^ while the stretching vibration of this siloxane bond corresponds to a wavenumber of 1060 cm^−1^.^50,51^ The peak at 550–650 cm^−1^ corresponds to the stretching vibration of the five-membered ring in JZO zeolite.^10,52^ During the 2 days induction stage, the peaks at 451 cm^−1^ and 586 cm^−1^ were broad and weak. Their gradual narrowing over time indicates the progressive ordering of disordered Si–O bonds. This trend emerged earlier and intensified more rapidly in seeded samples, suggesting that short-ordered building units formed more rapidly during early crystallization stages. This aligns well with XRD results. The combined characterizations of XRD, FTIR, and TEM collectively demonstrate that JZO seeds and fluoride anions exert a dual promotional effect on the nucleation and growth of JZO zeolites, expanding the synthesis window for this intriguing extra-large pore zeolite and thereby enabling a broader range of potential applications.

Additionally, we compared the XRD patterns of samples collected at 5, 7, and 9 days (Fig. S8), together with the SEM morphologies of samples obtained after 5 days of crystallization (Fig. S9), between seeded and seed-free samples with Si/Al ratios of 15 and 50. The PXRD results (Fig. S8) indicate that seed-assisted synthesis accelerates the crystallization process, leading to the earlier development of well-defined diffraction peaks. A noticeable difference in crystallinity is observed at shorter crystallization times (*e.g.*, 5 days), whereas at longer times (7–9 days), the crystallinity of seeded and seed-free samples becomes comparable, and in some cases, the seed-free samples exhibit slightly higher peak intensities. These results suggest that the primary role of seeds is to accelerate nucleation and crystallization kinetics rather than to enhance the final crystallinity. SEM images (Fig. S9) reveal distinct morphological differences between the seeded and seed-free samples after 5 days of crystallization, with the differences varying depending on the Si/Al ratio. At a Si/Al ratio of 15 (Fig. S9a and b), both samples consist of aggregated nanocrystals with polyhedral features. The seed-free sample (Fig. S9b) shows slightly increased aggregation and the presence of poorly defined small particles or irregular fragments, resulting in a less well-defined morphology. In contrast, more pronounced differences are observed at a Si/Al ratio of 50 (Fig. S9c and d). The seeded sample (Fig. S9c) exhibits aggregated nanocrystals with partially preserved structural features, whereas the seed-free sample (Fig. S9d) displays highly irregular aggregates composed of sheet-like or plate-like structures, indicating less controlled crystal growth and the possible presence of secondary phases. These results suggest that seeds facilitate the crystallization process by providing heterogeneous nucleation sites, thereby accelerating nucleation and influencing crystal growth behavior. This effect is more pronounced at early crystallization stages, while differences in final crystallinity are less pronounced and dependent on the synthesis conditions. N_2_ physisorption results (Fig. 3 and Table S3) further reveal that seeded samples exhibit increased specific surface area (by 1.1–4.2%) and pore volume (by 1.2–4.8%) compared to seed-free analogues, depending on Si/Al ratios. This improvement suggests that seeding facilitates the development of more regular and accessible pore networks, thereby enhancing diffusion properties.

### Catalytic performance

**Fig. 6:**
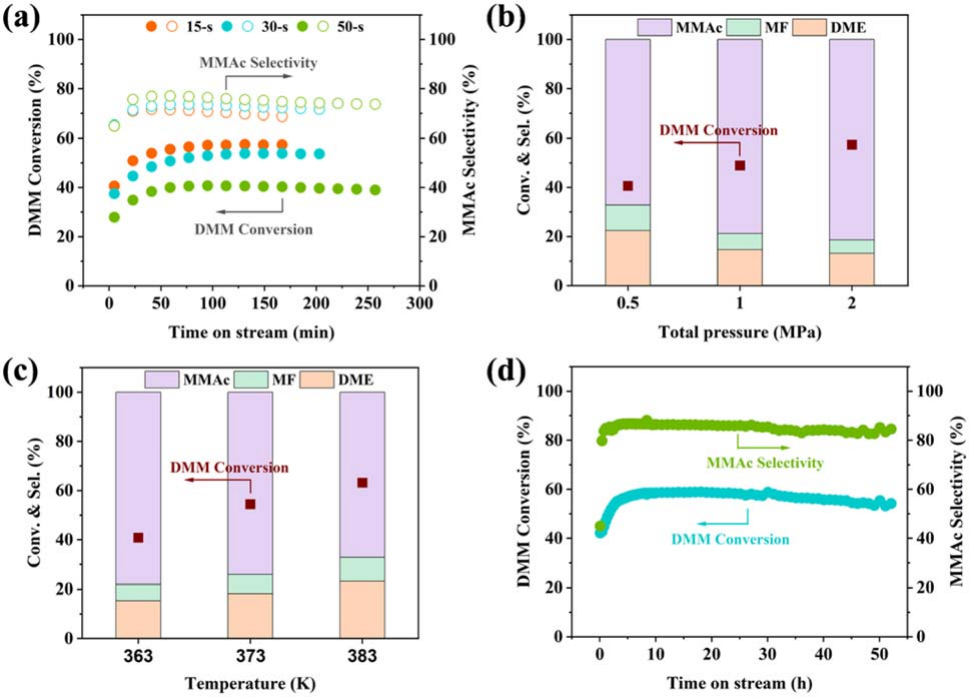
(a) DMM carbonylation over JZO with different Si/Al ratios. The catalytic performance of JZO with Si/Al ratio of 30 as a function of total pressure (b), reaction temperature (c), and the long time-on-stream test (d). Reaction conditions: (a) 0.05 g zeolite, *T* = 373 K, *P* = 1 MPa, *P*_DMM_ = 17.4 kPa, CO = 30 mL min^−1^, *n*_CO_/*n*_DMM_ = 57, WHSV_DMM_ = 1.98 h^−1^; (b) 0.05 g zeolite, *T* = 363 K, *P* = 0.5–2 MPa, *P*_DMM_ = 8.7–34.8 kPa, CO = 30 mL min^−1^, *n*_CO_/*n*_DMM_ = 57, WHSV_DMM_ = 1.98 h^−1^; (c) 0.05 g zeolite, *T* = 363–383 K, *P* = 1 MPa, *P*_DMM_ = 17.4 kPa, CO = 30 mL min^−1^, *n*_CO_/*n*_DMM_ = 57, WHSV_DMM_ = 1.98 h^−1^; (d) 0.1 g zeolite, *T* = 363 K, *P* = 2 MPa, *P*_DMM_ = 17.4 kPa, CO = 30 mL min^−1^, *n*_CO_/*n*_DMM_ = 114, WHSV_DMM_ = 0.98 h^−1^. JZO zeolites were synthesized with seeds and P-free.

Zeolite-catalyzed carbonylation of dimethoxymethane (DMM) to methyl methoxyacetate (MMAc) is a promising route for producing biodegradable polymers without the usage of noble metals and corrosive reagents under mild conditions.^53,54^ Previous studies have shown that catalytic performance in DMM carbonylation is strongly influenced by the interplay between pore structure and acid properties, where an appropriate Si/Al ratio is required to balance activity and selectivity.^55^ In this context, extra-large-pore zeolites provide a more open reaction environment and improve the accessibility of Brønsted acid sites. Consistent with this understanding, JZO zeolite has been reported to exhibit higher activity in DMM carbonylation compared with FAU zeolite.^11^ However, the previously reported JZO with limited Si/Al ratio of *ca.* 20 precludes the comprehensive investigation of its catalytic performance in DMM carbonylation. Here, JZO zeolites with varying Si/Al ratios synthesized by seed-assisted methods were tested following the procedures as described in a previously reported work.^11^ It is worth to note that dimethyl ether (DME) and methyl formate (MF) are the by-products originated from the DMM disproportionation reaction. P-free JZO zeolites (denoted as 15 s, 30 s, 50 s) showed high DMM carbonylation activity and selectivity at relative mild conditions (temperature of 373 K and total pressure of 1 MPa) (Fig. 6a). JZO (30 s) showed the highest MMAc yield with a DMM conversion of *ca.* 53% and MMAc selectivity of *ca.* 73%, which is comparable to or higher than values reported for conventional zeolites (*e.g.*, FAU, MFI, and MOR) under similar conditions in the literature.^55^ Therefore, JZO (30 s) was considered for further investigation, including the influence of reaction temperatures and total pressures. Increasing total pressures from 0.5 to 2 MPa with constant ratio of CO and DMM enhanced DMM conversion and MMAc selectivity simultaneously (from DMM conversion of 40.6% to 57.3% and MMAc selectivity of 67.2% to 81.4%) (Fig. 6b). Increasing reaction temperature from 363 to 383 K promoted DMM conversion (from 40.8 to 63.2%) (Fig. 6c). However, MMAc selectivity decreased from 78.1 to 67.2% due to the enhancement of DMM disproportionation. It is concluded that higher total pressure and moderate reaction temperature could achieve both high DMM carbonylation activity and selectivity. A time-on-stream (TOS) test of 50 h was performed at optimized conditions (Fig. 6d), *i.e.*, temperature of 363 K, total pressure of 2 MPa and WHSV of 0.98 h^−1^. DMM conversion decreased only slightly from 58.5 to 54.1% and MMAc selectivity remained above 84%, exhibiting excellent DMM carbonylation stability. The results demonstrate that the Si/Al ratios of JZO, *i.e.*, the adjustment of the spatial distribution of Al, could effectively modulate the activity and selectivity of the DMM carbonylation reaction, providing guidelines for developing industry-relevant zeolite-based catalysts.

## Conclusions

JZO zeolites with Si/Al ratios of 15, 30, and 50 were successfully synthesized *via* a fluorine-assisted route. Complementary characterization techniques were employed to systematically investigate the effects of synthesis parameters (*i.e.*, hydrofluoric acid concentration, water content, seeds, and Si/Al ratio) on the structural and physicochemical properties of JZO. Gel composition optimization experiments demonstrated that appropriate amounts of water and hydrofluoric acid facilitate the crystallization of JZO zeolites with tunable Si/Al ratios. SEM and acidity characterization (NH_3_-TPD, Py-FTIR, and ^27^Al MAS NMR) results revealed that with increasing Si/Al ratio, the morphology of JZO zeolite gradually evolves from well-defined polyhedral crystals to nano-platelet aggregates. Additionally, the total acidity of the materials decreases, and the strength of strong acid sites is notably weakened. Systematic analyses of products at different crystallization stages *via* XRD, TEM, IR, and N_2_ physisorption indicated that the crystallization rate and crystallinity of JZO are significantly enhanced by seed addition. Seed addition effectively suppresses impurity formation, while increasing the specific surface area and pore volume of the materials by 1.1–4.2% and 1.2–4.8%, respectively. In summary, both JZO seeds and fluoride ions exert significant regulatory and promotional effects on the nucleation and crystal growth of JZO zeolite. The JZO zeolites with different Si/Al ratios synthesized *via* a combination of the “fluorine-deficient” method and seed addition, were applied to the DMM carbonylation reaction to evaluate their catalytic performance. Results demonstrate that adjusting the Si/Al ratio of JZO zeolites to control the spatial distribution of aluminum atoms effectively optimizes both catalytic activity and target product selectivity in the DMM carbonylation reaction. JZO (Si/Al = 30) showed the highest MMAc yield with DMM conversion of *ca.* 58.5% and MMAc selectivity of *ca.* 84%, exhibiting excellent stability over a time-on-stream reaction of 50 h. These findings provide experimental foundations for the efficient synthesis and tailored application of JZO zeolites.

## Author contributions

Feng Shao, Peng Lu and Valentin Valtchev conceived and supervised the project. Shuman Gao and Xinxin Wang performed the zeolite syntheses, characterizations and data analysis. Shaolei Gao and Liang Qi carried out the DMM carbonylation tests, data analysis and interpretation. Mohammad Fahda collected the FTIR and solid-state NMR data. Haijun Yu, Dongyue Wang, Xiuning Liu and Zijian You performed the synthesis of OSDAs, conventional characterizations and data analysis. Feng Shao, Peng Lu, Valentin Valtchev, Shuman Gao and Xinxin Wang wrote the manuscript. All authors contributed to the discussion and revision of the manuscript.

## Conflicts of interest

There are no conflicts to declare.

## Supplementary Material

RA-016-D6RA01852A-s001

## Data Availability

The data that support the findings of this study are available on request from the corresponding authors. Supplementary information (SI) is available. See DOI: https://doi.org/10.1039/d6ra01852a.
