# Supplementary material for: Extra-large pore JZO zeolites with tunable Si/Al ratios as efficient catalysts for degradable plastic monomer production
Source: RSC Adv. 2026 Jun 1;16(32):29502–14. doi: 10.1039/d6ra01852a (PMC13227518; doi:10.1039/d6ra01852a)
Supplement: RA-016-D6RA01852A-s001 [file RA-016-D6RA01852A-s001.pdf]

Supplementary Information (SI) for RSC Advances.
This journal is © The Royal Society of Chemistry 2026

Extra-large pore JZO zeolites with tunable Si/Al ratios as efficient catalysts for degradable plastic monomer production

Shuman Gao‡[a, b], Xinxin Wang‡[a], Shaolei Gao[c], Mohammad Fahda[d], Dongyue Wang[b], Haijun Yu[a, b], Zijian You[b], Xiuning Liu[b], Liang Qi[b], Feng Shao*[a], Peng Lu*[b], Valentin Valtchev*[d]

[a]Key Laboratory of Marine Chemistry Theory and Technology of Ministry of Education, College of Chemistry and Chemical Engineering, Ocean University of China, Qingdao 266100, China.

[b]The ZeoMat Group, Key Laboratory of Photoelectric Conversion and Utilization of Solar Energy, Qingdao New Energy Shandong Laboratory, Qingdao Institute of Bioenergy and Bioprocess Technology, Chinese Academy of Sciences, 266101 Qingdao, China.

[c]National Engineering Research Center of Lower-Carbon Catalysis Technology, Dalian National Laboratory for Clean Energy, Dalian Institute of Chemical Physics, Chinese Academy of Sciences, Dalian 116023, Liaoning, China.

[d]Normandie University, ENSICAEN, UNICAEN, CNRS, Laboratoire Catalyse et Spectrochimie, F-14000 Caen, France.

‡co-first authors.

E-mail: feng.shao@ouc.edu.cn, lupeng@qibebt.ac.cn, valentin.valtchev@ensicaen.fr

Contents:

Supplementary Figures

Figure S1. Liquid phase ^{1}H NMR (a) and ^{13}C NMR (b) spectra of TCyMPI in $CDCl_3$.

Figure S2. PXRD patterns of as-made JZO zeolites obtained with different Si/Al ratios after heating for 5 days.

Figure S3. SEM images of as-made JZO zeolites obtained with a Si/Al ratio of 70 after heating for 11 days.

Figure S4. PXRD patterns of as-made JZO zeolites obtained with different Si/Al ratios over different crystallization times.

Figure S5. PXRD patterns of as-made JZO zeolites obtained with different Si/Al ratios after heating for 8 days.

Figure S6. PXRD patterns of as-made products obtained with different Si/Al ratios over different crystallization times.

Figure S7. TG (a) and DTA (b) profiles of as-made JZO zeolites obtained with different Si/Al ratios.

Figure S8. The XRD patterns of the cnw-JZO zeolite with Si/Al ratios of 15 (a) and 50 (b) after heating for 5, 7, and 9 days.

Figure S9. The SEM images of 15-s-cnw-5d (a), 15-n-cnw-5d (b), 50-s-cnw-5d (c) and 50-n-cnw-5d (d) after heating for 5 days.

Supplementary Tables

Table S1. Elemental analysis results of P-free JZO zeolites obtained with Si/Al = 70 using HF.

Table S2. Summary of synthesis and elemental analysis over samples obtained in hydroxide media.

Table S3. Textural properties of P-free JZO zeolites.

Table S4. Acid sites concentration and strength for cnw-JZO zeolites.

Supplementary Figures:

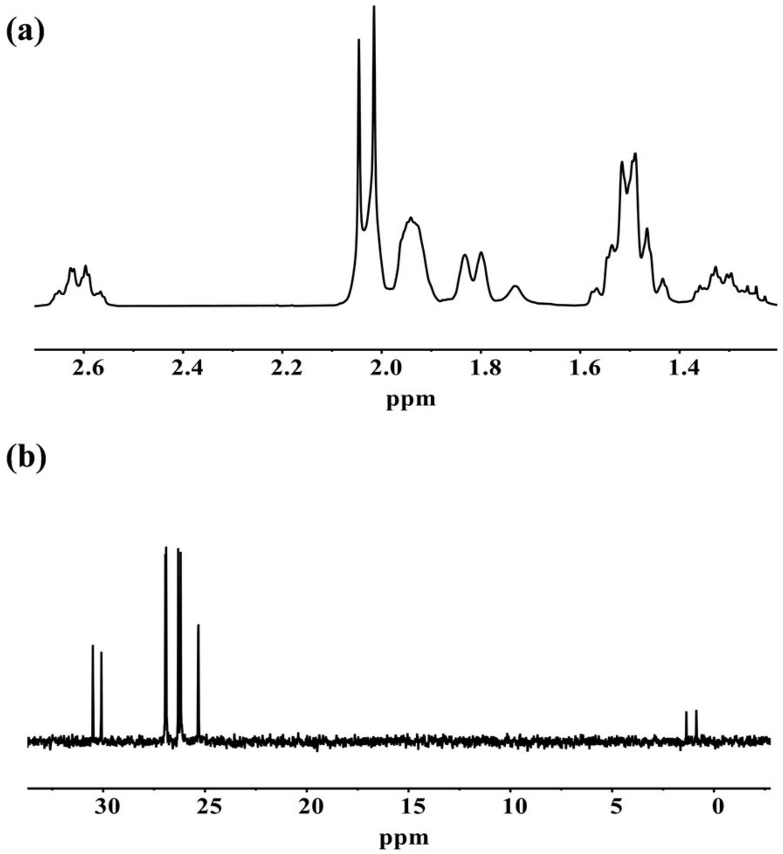

Figure S1. Liquid phase ^{1}H NMR (a) and ^{13}C NMR (b) spectra of TCyMPI in $CDCl_3$.

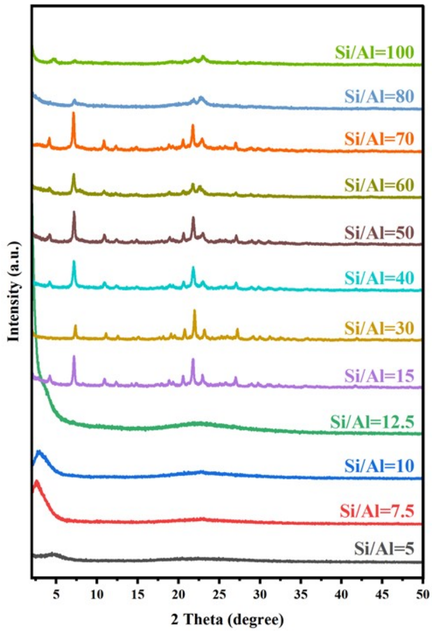

Figure S2. PXRD patterns of as-made JZO zeolites obtained with different Si/Al ratios after heating for 5 days. The gel molar composition is 1.0 SiO_2: 0.5 TCyMPOH: x Al_2O_3: 0.2 HF: 5 H_2O, 5 wt.% seed, with x = 0.100, 0.067, 0.05, 0.040, 0.033, 0.017, 0.013, 0.010, 0.008, 0.007, 0.006, 0.005 corresponding to Si/Al ratio of 5, 7.5, 10, 12.5, 15, 30, 40, 50, 60, 70, 80, 100, respectively.

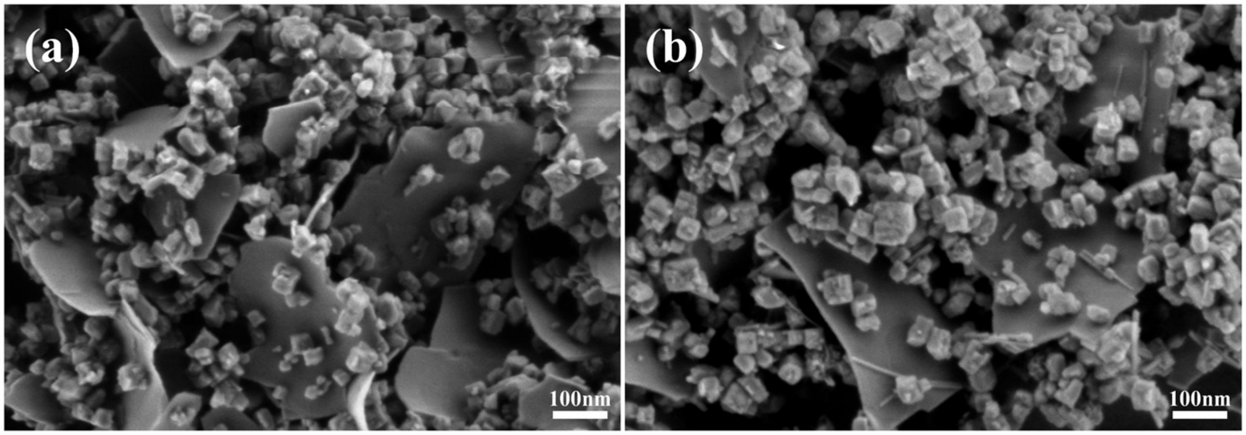

Figure S3. SEM images of as-made JZO zeolites obtained with a Si/Al ratio of 70 after heating for 11 days. 70-s-pre (a1), 70-n-pre (a2). The gel molar composition is 1.0 SiO_2: 0.5 TCyMPOH: 0.007 Al_2O_3: 0.2 HF: 5 H_2O, s-5 wt.% seed, n-no seed.

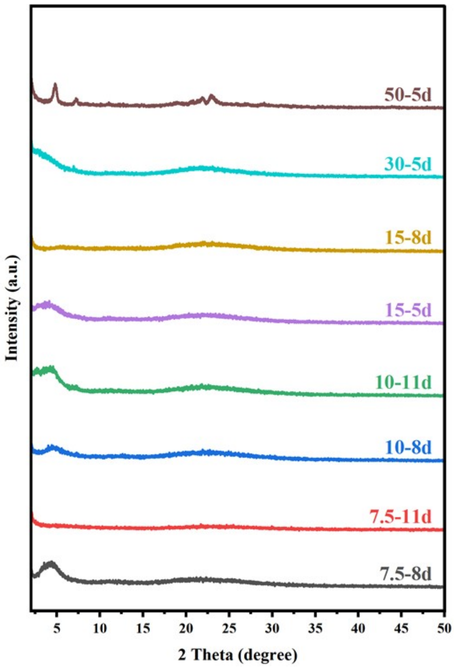

Figure S4. PXRD patterns of as-made JZO zeolites obtained with different Si/Al ratios over different crystallization times. The gel molar composition is 1.0 SiO_2: x Al_2O_3: 0.5 TCyMPOH: 0.2 HF: 10 H_2O, 5 wt.% seed, with x = 0.067, 0.05, 0.033, 0.017, 0.010 corresponding to Si/Al ratio of 7.5, 10, 15, 30, 50, respectively.

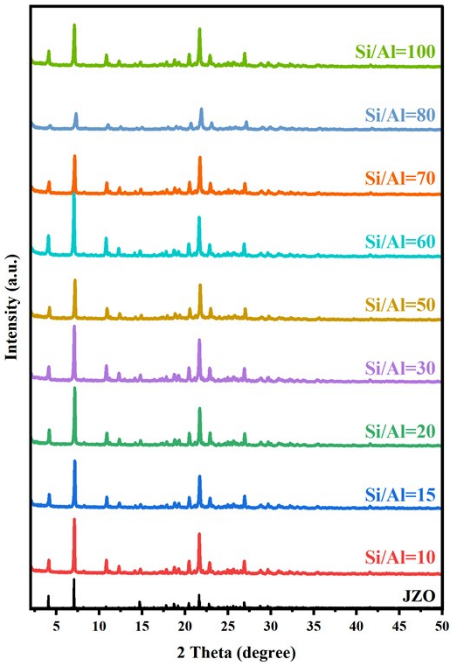

Figure S5. PXRD patterns of as-made JZO zeolites obtained with different Si/Al ratios after heating for 8 days. The gel molar composition is 1.0 SiO_2: x Al_2O_3: 0.5 TCyMPOH: 10 H_2O, 5 wt.% seed, with x = 0.050, 0.033, 0.025, 0.017, 0.010, 0.008, 0.007, 0.006, 0.005 corresponding to Si/Al ratio of 10, 15, 20, 30, 50, 60, 70, 80, 100, respectively.

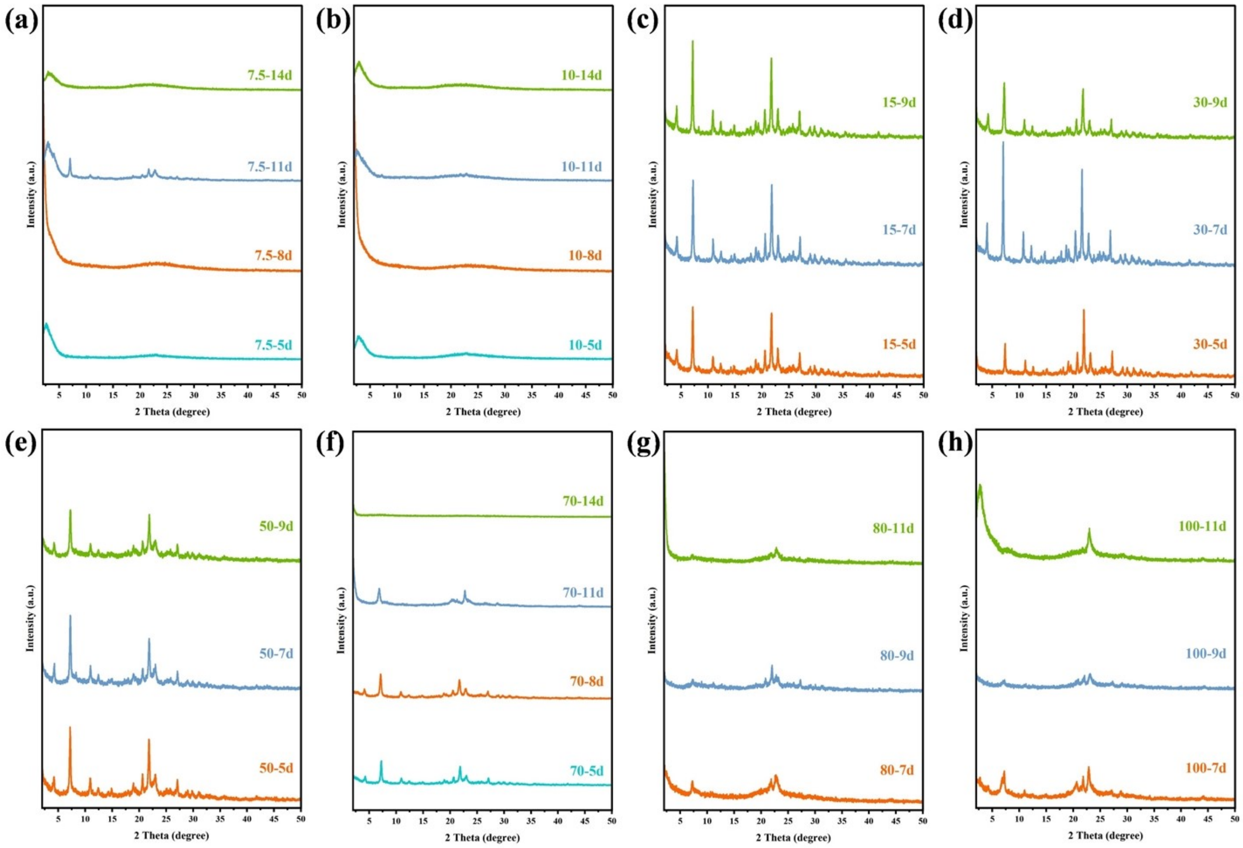

Figure S6. PXRD patterns of as-made products obtained with a gel Si/Al ratio of 7.5 (a), 10 (b), 15 (c), 30 (d), 50 (e), 70 (f), 80 (g) and 100 (h) over different crystallization times. The gel molar composition is 1.0 SiO_2: x Al_2O_3: 0.5 TCyMPOH: 0.2 HF: 5 H_2O, 5 wt.% seed, with x = 0.067, 0.05, 0.033, 0.017, 0.010, 0.007, 0.006, 0.005 corresponding to the Si/Al ratio of 7.5, 10, 15, 30, 50, 70, 80, 100, respectively.

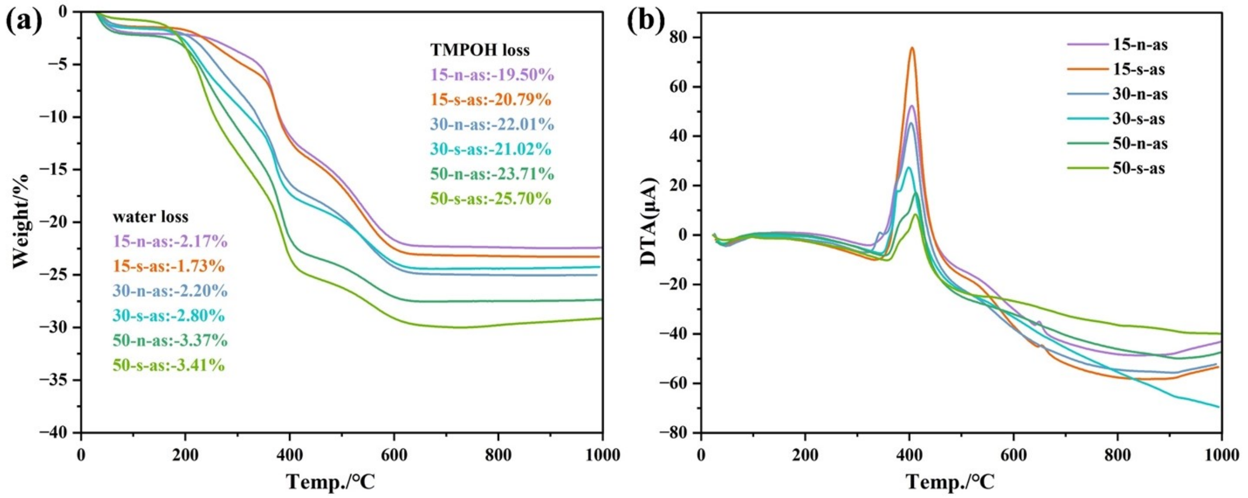

Figure S7. TG (a) and DTA (b) profiles of as-made JZO zeolites obtained with different Si/Al ratios. The gel molar composition is 1.0 SiO_2: 0.5 TCyMPOH: x Al_2O_3: 0.2 HF: 5 H_2O, with x = 0.010, 0.017, 0.033 corresponding to Si/Al ratio of 50, 30, 15, respectively. s-5 wt.% seed, n-no seed.

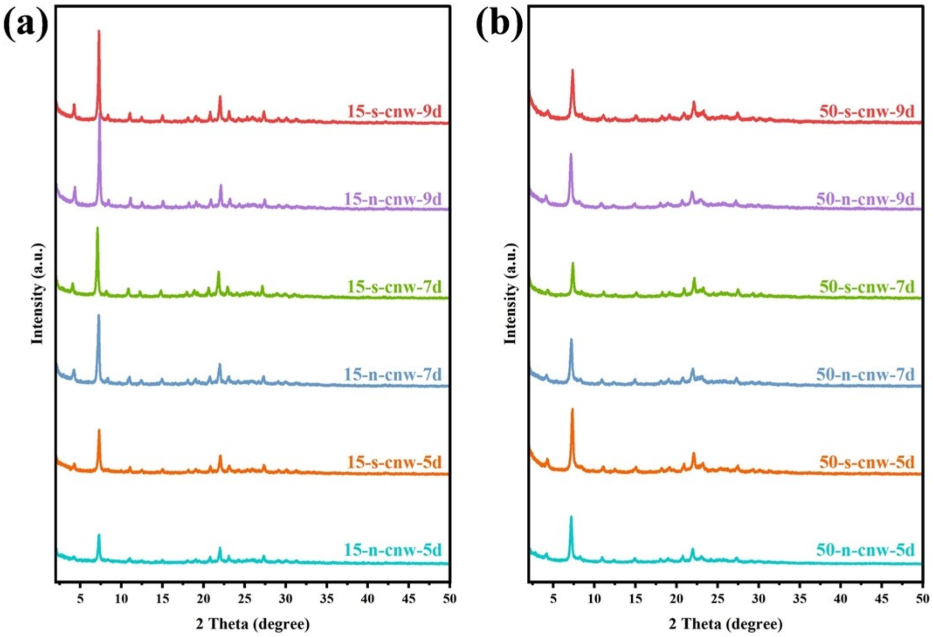

Figure S8. The XRD patterns of the cnw-JZO zeolite with Si/Al ratios of 15 (a) and 50 (b) after heating for 5, 7 and 9 days. The gel molar composition is 1.0 SiO_2: 0.5 TCyMPOH: x Al_2O_3: 0.2 HF: 5 H_2O, with x = 0.010, 0.033 corresponding to Si/Al ratios of 50, 15, respectively. s-5 wt.% seed, n-no seed.

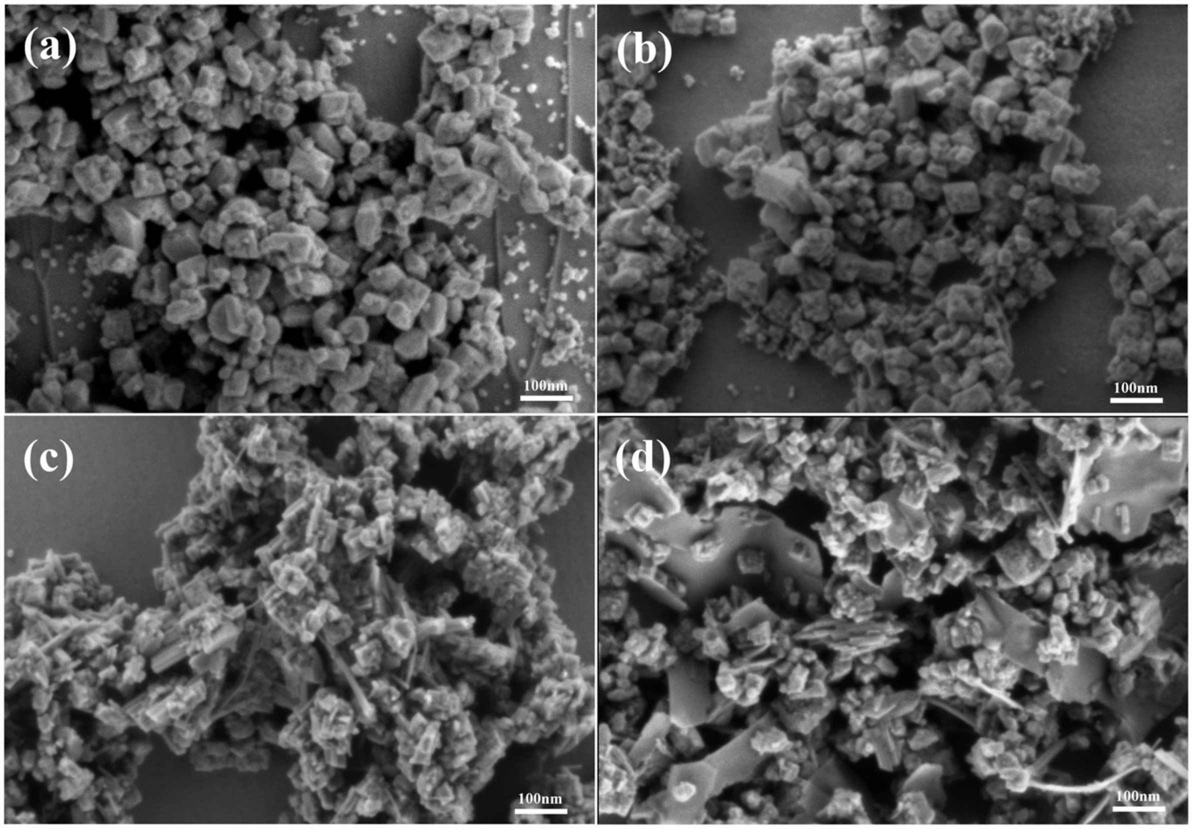

Figure S9. The SEM images of 15-s-cnw-5d (a), 15-n-cnw-5d (b), 50-s-cnw-5d (c) and 50-n-cnw-5d (d) after heating for 5 days. The gel molar composition is 1.0 SiO_2: 0.5 TCyMPOH: *x* Al_2O_3: 0.2 HF: 5 H_2O, with x = 0.010, 0.033 corresponding to Si/Al ratios of 50, 15, respectively. s-5 wt.% seed, n-no seed.

Supplementary Tables:

Table S1. Elemental analysis results of P-free JZO zeolites obtained with Si/Al = 70 using HF.

**Zeolite**	**XRF**		
	**SiO_2 %**	**Al_2O_3 %**	**Si/Al**
70-s-cnw	97.22	1.96	48.84
70-n-cnw	97.26	1.65	49.99

Table S2. Summary of synthesis and elemental analysis over samples obtained in hydroxide media.

**Entry**	**Si/Al**	**H_2O/SiO_2**	**Time (day)**	**seed**	**Phase**	**Yield (wt.%)**	**XRF**		
							SiO_2 %	Al_2O_3 %	Si/Al
1	10	10	8	5 wt.%	JZO	3.4	-	-	-
2	15	-	-	-	JZO	15	84.5	6.75	10.6
3	20	-	-	-	JZO	17.5	94.5	4.79	16.8
4	30	-	-	-	JZO	11.3	95.4	4.05	20.0
5	50	-	-	-	JZO	13	97.4	2.22	37.3
6	60	-	-	-	JZO	7	-	-	-
7	70	-	-	-	JZO	6	-	-	-
8	80	-	-	-	JZO	6	-	-	-
9	100	-	-	-	JZO	4	-	-	-

Table S3. Textural properties of P-free JZO zeolites.

Sample	S_{BET} (m^2/g)[a]	$V_{micropore}$ (cm^3/g)[b]
15-n-cnw	886.9	0.31
15-s-cnw	901.9	0.32
30-n-cnw	873.8	0.30
30-s-cnw	910.2	0.32
50-n-cnw	827.8	0.27
50-s-cnw	836.8	0.27

[a]Apparent surface area calculated using the Rouquerol BET criteria. [b]The micropore volume was obtained by N_2@77K adsorption using the t-plot method.

Table S4. Acid sites concentration and strength for cnw-JZO zeolites.

**Sample**	**Peak area**			**Adsorbed ammonia (µmol/g catalyst)**		
	**Peak1**	**Peak2**	**Peak area ratio**	**Weak acid**	**Medium-strong acid**	**Total acidity**
15-s-cnw	4543	14576	10: 32	47.14	255.33	351.35
30-s-cnw	3301	11853	10: 36	34.25	170.87	220.38
50-s-cnw	2060	7574	10: 37	21.34	78.59	99.93
15-n-cnw	5200	14311	10: 26	53.75	148.48	202.23
30-n-cnw	2885	14345	10: 50	29.93	148.84	178.77
50-n-cnw	2259	6003	10: 27	23.44	62.29	85.73
